# An open invitation to productive conversations about feminism and the spectrum of eating disorders (part 2): Potential contributions to the science of diagnosis, treatment, and prevention

**DOI:** 10.1186/s40337-022-00572-3

**Published:** 2022-04-19

**Authors:** Andrea LaMarre, Michael P. Levine, Su Holmes, Helen Malson

**Affiliations:** 1 School of Psychology, Massey University Auckland, Private Bag 102904, North Shore, Auckland, 0745 New Zealand; 2 Kenyon College, Gambier, OH USA; 3 University of East Anglia, Norwich, UK; 4 University of the West of England, Bristol, UK

## Abstract

The role of feminism in eating disorders research, treatment, and advocacy continues to be debated, with little agreement in sight about the role—or lack thereof—of feminist eating disorders work. In these debates, the opportunity to open fruitful conversations about eating disorders that generate new possibilities for researching, treating, and preventing them is missed. This article is the second in a series of two papers that invite such a discussion. In this article, we focus on five key contributions that feminist eating disorder work has made and can make moving forward. These are contextualizing treatment, attending to lived experiences, expanding the meanings of “sociocultural influences,” diversifying methodologies, and situating recoveries. We do not propose to offer a “final word” on feminisms and eating disorders, but instead to start conversations about how we understand, research, and treat eating disorders.

## Plain English summary

There continue to be debates about what role, if any, feminism has to play in eating disorders research, treatment, and advocacy. In these debates, we sometimes miss the chance to engage in productive dialogue about what the past and present of feminist eating disorders research, treatment, and prevention can offer—and where it might grow. This article, the second in a series of two papers that invite such a discussion, focuses on five key contributions that feminist eating disorder work has made and can make moving forward. These are: considering treatment in context, attending to lived experiences, thinking about the meaning of “sociocultural influences,” broadening our approaches to doing research, and considering recovery in context. We do not intend this work to offer a “final word” on the role of feminisms for eating disorders. Instead, we want to spark and continue conversations about how we understand, research, and treat eating disorders.

## Introduction

This is the second in a two-part series in which we invite readers to join us in the process of answering the broad question “What can feminism(s) offer the eating disorders field?” Part 1 addressed misconceptions about feminist approaches by articulating the assumptions and principles that make an approach “feminist.” In this paper we proceed to explore in more detail what feminist approaches can offer the scientific endeavor to understand, treat, and prevent the spectrum of eating disorders while minimizing harm to individuals and redressing pernicious practices such as weight stigma that have been deeply embedded in psychology, medicine, and other sociocultural systems. We note here, and will address later, the fact that a vast majority of feminist work on eating disorders revolves around anorexia nervosa, a significant limitation that needs to be addressed as we move forward.

In Part 1 [1], we situated ourselves^1^ and provided an overview of feminist approaches and their roles and uses in the ED field. Part one enabled us to think through some of the critiques that have been levelled and leveraged against feminist work, including that it is solely about “blaming men” and that it is politicized in ways that render it antithetical to “unbiased,” evidence-based, clinical science. In this regard we explored the feminist critiques of neo-Kraepelinian psychiatry and mainstream clinical psychology, and we identified areas of overlap in values and methods. Within our discussion of current feminisms (plural), we noted the limitations and potential harm done by some approaches that perpetuate a gender binary view and/or privilege the concerns of middle/upper class White cisgender women. Consequently, and in light of substantial evidence that people from marginalized groups experience eating disorders and disordered eating [e.g. 2, 3], we illustrated the importance of, and challenges in, a perspective that seeks to understand, respect, and work with diversities at the intersections of gender, race, class, sexual orientation, disabilities, and so forth.

Thus, in Part 1, we argued that feminist approaches, in being attuned to power-inequalities, invite us to take contextualizing and empowering approaches to research, advocacy, and treatment. To emphasise that point we considered one of critical feminism’s key contributions to the eating disorder field—its unpacking of diagnostics by situating distress within sociocultural contexts that hold (i.e., restrict) certain bodies to particularized and narrow standards.

In Part 2 we highlight five more areas in which feminist approaches to eating disorders offer insights that might be leveraged to address problematic areas in the eating disorders fields, including omissions and tenacious inequities. These are contextualizing treatment, attending to lived experiences, expanding the meanings of “sociocultural influences,” diversifying methodologies, and situating recoveries. We do not address prevention in detail here because there is a substantial body of literature elsewhere that reviews the theories, methodologies, and findings of feminist approaches [4–7].

## Contextualizing treatment

Feminist work has the potential to support empowering and impactful therapeutic work with those struggling with an eating disorder. Many approaches to treating eating disorders that have been accorded the seal of evidence-based approval, including family-based treatment, are actually based in some elements of feminist therapeutic approaches [8], with more or less acknowledgment in different times and places. For example, Lock [8] explains that “the process of empowering the family to find solutions to the problems that AN is causing is based in the nonauthoritarian stance of Milan systems therapy (Palazzoli, 1973) as well as feminist theory” (p. 276). Thus, it is surprising to see how recognition of explicitly feminist approaches has been largely absent from discussion of “mainstream” eating disorders treatment over the past several decades or has been reduced to a particularized version of what feminism is or does.

Early feminist therapists/authors blended traditional psychodynamic approaches with a “narrative of political empowerment” [9] and specific attention to sexual victimization [10], all the while harnessing the idea of the “personal as political” which was so fundamental to feminism, particularly in the late 70s and early 80s [11–15]. As early as 1993, Bordo described the excitement and challenges associated with bringing feminist approaches to the fore within a field already dominated by biomedical approaches, writing about a conference that was intended to spark a “breakthrough" for feminist approaches to eating disorders treatment. When the edited volume *Feminist Perspectives on Eating Disorders* [16] was published the next year, that breakthrough appeared imminent to some.

Nearly 30 years later that potential has not really come to fruition. This outcome reflects a number of complex historical and political factors, including the dominance of patriarchal structures and the hegemony of a narrow, biopsychiatric view of “science.” In a landscape of eating disorders treatment characterized—in part due to budgetary constraints—by broader trends toward short-term efficiency, reified as “evidenced-based” “managed care” [17], there is seemingly little room outside of private clinical practices for treatment approaches that deviate from particularized versions of the types of protocols and evidence required. The increasing value placed on evidence-based treatments has tended to privilege more “objectivist view[s] of science” [18, p. 490].

There are, of course, still feminist therapists working in eating disorders treatment today, as well as treatment centres established on firmly feminist grounds, although explicitly feminist-oriented eating disorder treatment centres are rare, particularly in the UK and North America. In 1976, in the UK, Susie Orbach co-founded and established the Women’s Therapy Centre (WTC), which seeks to place and treat eating disorders within the context of women’s cultural experiences [19]. Orbach’s legacy has been expanded in New York into what is now the WTCI: An Intersectional Feminist Psychotherapy Institute. For over 30 years The Renfrew Centre in the USA has similarly offered and indeed publicly marketed “feminist” residential and outpatient treatment programs for women.

Very little empirical data exist on the extent to which feminist questions of gender, and other intersecting factors, are substantially or effectively (or even nominally) addressed within clinical contexts. Many programs purport to focus on the needs of women. However, they do not publicize their approach as “feminist,” nor do they advertise any details about feminist components such as developing a critical social consciousness in explorations of the construction and enforcement of gender roles. What scant evidence exists indicates that these issues feature very little in the training of health professionals working in eating disorders [20] and in people’s experiences of treatment—despite clients explicitly saying that such a focus would have been helpful [21–23]. Some qualitative work, such as a study conducted by Holmes et al. [22], illustrates the potential for feminist approaches in ED treatment to situate EDs within sociocultural milieu and potentially minimize self-blame around the development of an ED. However, there is a paucity of research specifically exploring the applied potential of feminist treatment approaches or of feminist components of broader treatment programs. Further, and as discussed in Part 1, the treatment and research funding landscape is increasingly dominated by cognitive-behavioural and biomedical approaches to EDs. Thus, the sociocultural issues at the heart of feminist psychotherapy are treated as secondary or facilitating factors whilst “an undue emphasis on individual pathology” persists [24, p. 389].

Patients, however, have reported wanting to address and explore issues of gender and sexuality in treatment, only to find that such requests may be dismissed or ignored due to therapist resistance, inadequate training, and/or insufficient expertise [21, 25]. Where the evidence base of biopsycho*social* influences is addressed in treatment, this tends to be specifically around body image work or aspects of media literacy, perpetuating the idea that those experiencing eating disorders are somehow more ‘vulnerable’ media consumers [20]. This limited and potentially problematic focus leaves considerable room for exploration of the broader sociocultural factors (including not only the sexist construction of gender/roles, but also racism, ableism, heterosexism, transphobia, sizeism, and more) that shape dis/embodied experiences in the world. This is a particularly pressing need in light of the increasing recognition of the complexity and non-binary nature of gender, and of data indicating that gender diverse people have unmet needs within ED treatment [25, 26]. Scientist-practitioners and treatment/recovery advocates clearly need to make space to explore gendered experiences within and beyond treatment contexts.

Feminist work that carefully analyzes existing eating disorders treatment provides insights into the places in which feminist approaches can offer significant improvements in the delivery and evaluation of care. Eating disorder treatment “as usual” has been critiqued in the feminist canon for inscribing rules around food that are as rigid as those adopted in eating disorder practices [e.g., around timing and content of meals; 27]; generating adversarial rather than therapeutic relationships [28]; eclipsing the voices of those with eating disorders [29]; occluding the broader systemic factors that scaffold body distress (including racism, sizeism, and other “isms”; [30]); and invalidating the person in treatment as a fully human agent with the ability to envision alternative possible subjectivities [17, 31]. Knowing, as we do, that the efficacy of current treatments is limited and that there is all too often a “mismatch between therapeutic interventions and what people with anorexia [and other eating disorders] say about their experiences” [32, p. 188], why do we continue to silence feminist insights about eating disorders treatment?

Feminist scholarship, and in particular post-structuralist feminist work, tends to be critiqued for not proposing concrete “solutions,” specifically for focusing instead on deconstruction—how language and conceptual systems shape meanings, while tending to privilege certain groups as having the authority to speak, write, and influence. However, a close reading of feminist critiques reveals productive directions for treatment informed by a feminist lens. We begin our consideration of some of these critical analyses by exploring what taking a feminist approach to treating eating disorders does *not* mean.

Specifically, in our view, feminist approaches should *not* mean designing any of the following: treatment only for women or treatment that vilifies men; treatments that exclude cis men, trans men or women, or non-binary people with eating disorders; interventions focusing only on body image and media objectification of bodies; programs that intentionally employ only women as treatment directors or staff; or those ignoring a restoration of nutrition. On the contrary, feminist propositions for treatment include: 1. “shifting clinical interventions toward contextual variables” [32, p. 189] in order to focus on the structures, systems, and power differentials people face in their lives; 2. “expand[ing] our understandings of treatment success beyond weight restoration, medical stabilization and behavioural symptoms” [17, p. 326]; 3. “listen[ing] to people with eating disorders” [17, p. 326]; 4. re-imagining and expanding modalities for treatment to incorporate broader means of belonging and being in the world (e.g., supportive psychotherapy, narrative therapy, recovery model; [31]); and 5. “recognizing the differences between individual patients and respecting the meanings they attach to their illness” [28, p. 92]. Practically, this may look like embracing collaboration in clinical settings. Specifically, collaborating means recognising that, while a person with an ED might not always be acting in their own best interest, decisions made about their treatment can involve them in the process as humans. As is the case for research participants in clinical science, people with eating disorders have the right to informed consent and to transparency as decisions are discussed with and explained to them. Given that collaborative and individualized care are associated with improved outcomes (e.g., [33]), taking such an approach seems particularly promising.

From a feminist perspective, collaborative care necessitates a centring of the individual’s experience. Doing this meaningfully requires a deep and tightly held commitment to working with the person in the context of a respectful relationship that emphasizes power-sharing wherever possible [34]. This strongly supports and indeed extends the patient-oriented stance so clearly spelled out in various national treatment guidelines such as those issued by the UK’s National Institute for Health and Care Excellence (NICE; [35]). However, this tenet also highlights the current contradiction between, on the one hand, the absence of any reference in the NICE guidelines to consideration of interpersonal, sociocultural, and systemic factors as a part of treatment and, on the other, (a) an enormous body of evidence pointing to the importance of the person-professional relationship in treatment [36]; (b) compelling evidence supporting the operation of sociocultural factors in the development and maintenance of eating disorders and disordered eating [37]; and (c) a broader acceptance in the field that the causes, features, and treatment of eating disorders are “biopsycho*social*” in nature [37]. Once again, we see that what a feminist approach is really challenging is a dogmatic commitment to valuing only some kinds of evidence, produced by only some “authorities,” when determining what is “evidence-based.”

## Attending to lived experiences

Extending the theme of what qualifies as “evidence-based,” at the outset of this section it is important to acknowledge that simply hearing and perhaps quoting participant stories in the context of a research study with a “qualitative” component does not in and of itself constitute a fundamental shift to *valuing* the lived experiences of people with eating disorders or their families. While much feminist work has been qualitative in nature and may aim to “give voice” to lived experiences, almost all research studies are conducted in a way that continues to privilege the interpretation of the researchers and thus arguably gives little back to the participants [34, 38]. Qualitative work in general, and feminist qualitative work in particular, can, however, offer at least access to potentially empowering *interpretations of* participants’ voices. Notably, although feminist research can be qualitative*,* quantitative *or* mixed methods, the “feminist” aspect refers to the “political commitment” involved in the process of exploring and ultimately transforming oppressive systems (39, p. 28). Qualitative research is not in and of itself feminist, unless the authors claim and work with a feminist ﻿﻿﻿﻿theoretical and methodological lens﻿﻿﻿, ﻿working with and through the gendered dynamics of power (typically enmeshed with other axes of power) involved in the issues they are analyzing—and in the research process itself.

The distinction between “giving voice” and recording voices in eating disorder research requires consideration of how the voices of those with eating disorders have typically been imagined. Across different treatment modalities, from family-based treatment to narrative therapy and beyond, “the voice of the eating disorder” is commonly dissociated from the person, through a process of externalization, often by consensual agreement between the therapist and the person. Shades of a triangulated “eating disorder voice” in addition to those of the patient and therapist can be traced to the writings of Bruch and others in the late 1970s and 1980s. Lived experience descriptions of being controlled, and indeed tormented, by such a voice are found throughout the eating disorder treatment literature [40]. As with most topics in eating disorder research, the “voice” has been explored mostly in the context of restrictive eating disorders and is sometimes referred to as the “anorexic voice” or “Ed” (short for the “*e*ating *d*isorder” voice; [41–43]. The prevalence and personal relevance of “the voice” within eating disorder stories has led to efforts at developing scales to explore how this voice is tied to selfhood amongst people diagnosed with anorexia [41].

The concept of “the eating disorder voice” potentially enriches our understanding of what it means to be a person *with* an eating disorder [41, 42, 44]. However, it is not without its critics [29], and is unlikely to completely explain the subjective experience of “having” or “living with” an eating disorder. There is also a danger in assuming that “the voice” is a universal experience. Feminist literatures can be helpful in unpacking the complexity inherent in such a concept, thereby helping us to avoid theoretical and empirical constructions that *subsume identity and eating disorder together* and those that *impose structures of meaning onto people’s experiences.*

A look at feminist eating disorder literatures reveals how viewing people as *being* their eating disorders (e.g., by calling them “the anorexic”) may limit  a person's ability to re-imagine non-eating disordered subjectivities [31]. We acknowledge that externalizing the “eating disorder voice” allows for a separation that enables productive exploration of their subjectivity outside of the narrow, if not suffocating, confines created by cycles of narrow and obsessive rules, self-monitoring, self-criticism over inevitable failure, and the resulting eating-weight-shape-related distress. Indeed, this capacity for re-visioning subjectivity and re-writing the story of one’s life lies at the heart of narrative approaches to treating eating disorders, which arguably dovetail with feminist work in this realm.

Michael White’s [45] reflections on using externalization in narrative therapy for anorexia nervosa in particular emphasize the importance of working with stories, however incomplete or chaotic, authored by the person, rather than imposing categorical labels on the voices that emerge in these stories. Situating the person with lived experience as the expert in the construction, expression, and revision of their own story becomes incredibly important here. White [45] notes the heterogeneity within experiences of eating disorders, arguing for the thoughtful use of externalization driven by the person, rather than the imposition of a particularized version of that voice.

This person-driven perspective on externalization of “the voice” is important in light of critiques of the ways in which, just like subsuming people’s identities into “the anorexic” might limit possibilities for re-writing the self [31], so too might imposing an artificial separation of self and eating disorder [29]. Feminist perspectives allow for engagement with how “both” or several voices—those of the person experiencing eating distress negotiating their subjectivity within and beyond this distress and with the “eating disorder voice”—are speaking within broader sociocultural contexts consisting of multiple and often conflicting discourses about “good,” “bad,” “healthy,” “unhealthy,” “in control,” and “out of control” subjectivities. Paula Saukko’s [29] book *The Anorexic Self* deals quite explicitly with such dilemmas. Rather than assuming that either voice (that of “the person within” or “the eating disorder”) is “true” or “correct,” an approach such as Saukko’s [29] invites consideration of “the social nature of experiences and their interpretations” (p. 79).

Thus, a feminist lens enables consideration of how, in its deployment in clinical settings, a focus on “the eating disorder voice” may insufficiently address the confluence of different aspects of the multiple “voices” or discourses involved in constituting subjectivity [29, 46]. An exclusive focus on “Ed” is also fundamentally at odds with a critical feminist interpretation of the entwined nature of social inequities, power differentials, and other structural factors that are dis/embodied in eating and body distress.

In popular forums, presenting the person experiencing this distress as being “ruled” by their eating disorder may lead to unintentional constructions of people with eating disorders as little more than a “cultural dupe” [47] or even as possessed by an invading tyrannical force. Feminist approaches, and sociocultural work in general, perhaps because they challenge “illness” constructions and the hegemony of biological reductionism, are often critiqued for somehow making eating disorders out to be easily changed issues of vanity or misguided choices. In reality, feminist approaches have never maintained this position and, more important, allow us to *explore that construction*, and in the process illuminate the ways in which we all are wrestling with similar and diverse sociocultural forces that shape our understandings and experiences of bodies, desire, emotions, identity, control, and self-in-relationships.

In this regard, we have long known that, like most or all people, people with eating disorders are conscious of how they are perceived, including how people perceive them in relation to their eating disorders or an idea of what an eating disorder “is” [48]. Engaging with feminist—particularly critical feminist—work on eating disorders invites us to scrutinize not just the ongoing psychological processes *within* a person but how various people constituting the eating disorders “field” (e.g., professionals, people seeking help, families) interact with others and their surroundings to construct particularized—and not always helpful—versions of self and subjectivity. The fact that eating disorders often develop during adolescence and early adulthood, when individuals’ sense of themselves, their gender, and their sexuality will likely be undergoing rapid change and when others’ perceptions of them will similarly be shifting, make this feminist attention to multiple, shifting, contextualised perceptions and voices particularly pertinent. Practically, a feminist perspective on voice and externalization invites an avoidance of *assuming* that “the voice” or “Ed” is an approach that all will resonate with—or that it is primarily internal and individual, that is, “intrapsychic.” Instead, a feminist perspective encourages the person to re/construct (re/author) their own story—and thus their own self—in relation (or not) to their own contextual *and* individual experiences [45].

## Expanding the meanings of “sociocultural influences”

Feminist scholars [6, 24, 49] have led the way in expanding our conceptions of what “sociocultural” means in terms of influences on body image, weight and shape concerns, and the spectrum of disordered eating. Their work has taken us far beyond “the media,” “sex roles,” and “peer teasing.” In this regard, engaging with the sociocultural surround—the discourses of gender, class, dis/ability, race, sexualities, achievement, and so forth that impact us and that we continue to construct through interaction—allows us to explicitly pluralize “the” eating disorder voice or “the” eating disordered experience. Effectively, taking a critical feminist stance toward lived experiences means that those experiences and their “influences” will never be assumed to be singular, especially in ways that are contained in and constrained by a diagnostic, treatment, or prevention manual [6].

In their critical feminist analysis Nasser and Malson [50] maintain that eating disorders are “culturally embedded, complex and heterogeneous collectivities of discursively constituted subjectivities, experiences and body management practices that can be read as expressing a variety of often gender-specific cultural norms, values and dilemmas” (p. 74; see also [51, 52]). This means that they cannot be extracted from their sociocultural surround, even if we believe our “general linear models” of statistical analysis are doing just that. The point here is not that the sociocultural surround, as a set of factors to be studied apart from an individual, is always solely and causally related to development of eating disorders. Rather, our understanding of how to prevent, treat, and even research them *must* contend with the determinants of how social, political, and economic power flows in, through, and around us all. Attending to these power dynamics and people’s individual circumstances within treatment, prevention, research, and advocacy settings means making space for people’s differences *and* their similarities to be named and honoured.

Certainly, we may observe and reasonably apply patterns in what people say about their eating disorders and how people articulate their experiences of recovery. However, finding a singular truth about “the lived experience” of an eating disorder is an impossible exercise. A critical feminist approach invites us to acknowledge and honour diversity (both within and across the various categories of eating disorder) as we consider how these articulations will always draw in part on the cultural repertoires people use to understand and articulate themselves. Moreover, the research encounters in which these articulations are generated and shared will themselves be laden with power [21, 53, 54]. It is this construction of research that we contend with in the next section.

## Diversifying methodologies

Fundamental differences in thinking about and applying research methods contribute significantly to the chasm between much of the critical feminist eating disorder literature and eating disorder literature “in the mainstream.” When critical feminist work uses methodologies that are not legitimized in certain contexts (e.g., academic journals), this ostensibly “unscientific” work is desk-rejected or subject to peer reviews which ask the work to be put into boxes (e.g., “exploratory”) into which it does not sit easily or, arguably, belong at all. The role of qualitative research—which has structured much but not all of feminist work—has been a key issue here. This section carefully examines the positionality and contributions of critical feminist approaches to methodology in order to foster new ways of engaging in eating disorder research.

The contention that all research is situated within a power-laden, politicized set of sociocultural forces means that there are legitimized, socioculturally specific ways of “seeing and doing things,” which are practiced by recognized professionals (“experts”) working in prestigious hospital/academic settings. If we accept this argument, or at least are committed to exploring and learning from it, we are on our way to engaging with critical feminist approaches to research. There are critical feminist roadmaps for how to go about this, though their signposts may be less explicit than those stemming from either positivist approaches (strictly empiricist) or post-positivist critiques of bias and politics in science.

A significant majority of the research in medicine and the social sciences on eating disorders is quantitative and/or based in positivist and post-positivist paradigms. These studies set out hypotheses (in some instances derived from a theory, and always already understood to be grounded in null hypotheses) about eating disorders. The researchers then engage in data collection and analytic methods that seek verification, that is, to determine whether or not these null/hypotheses are likely to be “un/true.” These approaches to eating disorders research typically presume that 1. there is a set of objective criteria emerging from the empirical data that will reveal and/or verify whether or not someone has or has had a “legitimate” eating disorder; 2. any number of other theoretically or practically relevant variables might be similarly defined and measured in order to subject their relationships with “legitimate” eating disorders to falsification/verification; and 3. therefore, the predicted “real” relationships between these variables can be illuminated, leading to “facts,” and hopefully to principles, if not laws, much like those in physics and chemistry.

This distillation does not in any way minimize the importance and sophistication of various research techniques used to explore eating disorders. Nevertheless, the basic premise that underlies this approach, typically configured as “mainstream”, is that some truths or ways of knowing are “objective” and move us in the direction of compiling “scientific facts.” Therefore, in our most definitive studies we must do everything we can to control for “contaminants” that would lead to a lack of clarity and cogency in the interpretation of the findings, thereby disrupting the overall direction of progress.

Taking a critical feminist stance on eating disorder research often (but not always) rejects these “scientific” truisms. Poststructuralist feminist eating disorder work in particular operates on the assumption that singular, absolute “truths” do not exist, and that research is embedded within and is thus influenced by the very systems it seeks to explore and/or critique. This approach often does away with the assumption that diagnostic criteria are objectively measurable manifestations of an underlying disease entity. Thus, research based on that assumption is questioned in terms of whether it has “actually” said anything about “real” eating disorders. This critique of a narrow application of what constitutes “science” is, not surprisingly, a major source of resistance to feminist work on eating disorders.

However, given what we know about barriers and exclusions that keep people from obtaining diagnoses of and treatment for eating disorders [55–57], can conclusions based only on people legitimized by the medical-psychiatric establishment as “truly having eating disorders” be taken as representative of those with eating disorders in general? After all, it was not that long ago that most “credible” (“real”?) psychiatrists/physicians and neuroscientists accepted as a given (a “scientific fact”) that males could not develop “hysteria” (conversion disorders), that homosexuality was a “perversion” of psychosexual development, and that vigorous exercise, including organized athletics, was a severe threat to the well-being of young women. Consistent with the operating principles of a fundamental science such as physics, taking a critical feminist stance encourages us to expand the science of eating disorders by interrogating the paradigms and resulting limitations in methodologies used in much eating disorder work and then proposing alternatives that invite more expansive and situated analyses of "eating disordered"  experiences, including behaviours. Of course, even some poststructuralist feminist work on eating disorders is conducted with those who *have* received a diagnostic label for their eating distress. It is still possible, in this research, to invite participants to share their experiences (including “outcomes”) *in relation to* how they were framed and cared for, for instance, in eating disorder treatment [28, 31, 58, 59].

There are numerous ways of engaging in eating disorder research within a critical feminist perspective. Over the course of 30 years feminist scientist-practitioner Niva Piran has written and spoken extensively about her participatory-relational-empowerment approaches to prevention and to research on female development. This network of theory and methodologies features traditional academic forms of quantitative and qualitative studies, while it operates well outside *standard* positivist thinking and designs for constructing and evaluating prevention and for thinking about general and specific risk factors [6, 7, 60]. Saukko’s [29] feminist research seeks to redress the replication of “social scientific conventions of analysis and writing” (p. 82), instead presenting “layered accounts” of experiences of eating distress. Like Piran, Saukko invites consideration of lived experiences in relation to participant critiques of dominant discourses on eating disorders, as well as exploring her own experiences and interpretations in her work.

LaMarre and Rice [61] worked with participants in eating disorder recovery to make short films, similarly allowing all concerned to work on/out not only what people articulate about their own recoveries but also how they engage with the concept of recovery as represented in the mainstream. Holmes [20] also investigated existing representations of eating disorder recovery, exploring these in relation to discourses on YouTube and in the scientific literature, and in recognition of her own subjectivity. Saunders and Eaton [62] conducted a photovoice exploration in which they invited perspectives on eating disorder recovery, applying what they found to lobby for change.

These studies openly engaged with the “stuff” of qualitative analyses in interaction with researcher subjectivities. Levy, Halse, and Wright [63] took this a step further, explicitly interrogating the epistemological lenses they were using in their work to think through different ways of finding meaning in their data. They discuss the challenge of examining qualitative data from participants diagnosed with eating disorders to identify patterns in discussions about health, food, and body image in relation to broader social discourses. Instead of seeking *coherence* in responses, Levy et al. [63] applied post-qualitative approaches to research, using Barad’s [64] ideas about intra-activity and Deleuze’s [65] concept of the “event.” Taking a stance of curiosity, the authors looked at the relationships between questions asked, participants’ responses, and expected/unexpected discourses.

This and other examples of engagement with interviewing and other research encounters as more than a simple exercise of systematically asking, answering, and sorting responses into intelligible truths, offer new ways of thinking. These methods can produce information, ideas, and insights that do not “fit” within dominant understandings, while improving the researcher-participant/patient dynamic. And, perhaps because the “findings” that result are “uncontrolled” and “unfit” in the classical sense, they contain important potentials. They can transform in fundamental ways what “we” (researchers, clinicians, patients, families, bloggers, journalists, etc.) “know to be true” about eating, bodies, and health [63], expanding and making more equitable the “we” while multiplying the truths in productive, socially just ways. Feminist principles and methods can broaden the set(s) of people to whom the facts apply and for whom the knowledge is helpful.

In addition, there is a tradition from the earliest days of feminist writings and beyond [12, 21, 46, 66] of authors including aspects of personal experience of eating/ body distress. This is entirely in keeping with the emphasis on the personal nature of political experience in feminist research and the mandate to reflect on the author’s own subjectivities and positionality. Despite the sense that the voices of those with lived experience of eating problems are valued in eating disorder research, such personal connections are often seen as entirely untenable in mainstream approaches and publishing arenas because they compromise the ideal of scientific “objectivity.” Indeed, more than one of us has had our qualitative work desk-rejected by mainstream eating disorder journals, and assertions in peer-reviews of our work that “it strikes the reviewer as very odd that the author would refer to personal experience” have not been uncommon.

We emphasize that, even as feminist research methods advocate for broadening and deepening the expanse of “evidence-based” knowledge, there is no requirement for all researchers and clinicians to abandon working suppositions about what constitutes a “true” eating disorder or even certain criteria for “good research.” Rather, what critical feminist studies of eating disorders tend to hold in common are four themes that we see as productive for eating disorders research more broadly and as fundamental to both research ethics and scientific humility. The first is the need for self-critical and public reflection on the power involved in all research processes. Second, it is crucial to consider, over and over, who is encouraged to speak up and get involved in research and who is not, by assessing the awareness people in general and people with eating disorders in particular have about what constitutes a “real” eating disorder. Third, and along the same lines, researchers, whether or not they are also clinicians—and especially if they are educators and supervisors—have an ethical and professional obligation to keep exploring their own subjectivity and assumptions about eating disorders and the people who experience them. Finally, to promote the embodiment of people with eating disorders and other research participants as people with the same kinds of needs and anxieties as we have, it is important to design and conduct research that collaborates with participants and produces knowledge that *truly* “gives back” to them in ways that result in positive personal, professional, and social change.

## Situating recoveries

Continuing with the thread of situating eating disorder research and its findings within a sociocultural surround, we now consider how feminist eating disorder work invites a consideration of “what we are aiming for” in eating disorder treatment. This aspect of the feminist paradigm is very important because, even within more mainstream approaches, eating disorder recovery is ill-defined beyond a general agreement that recovery constitutes symptom remission plus something more [67, 68]. Feminist work on eating disorders invites us to consider the “something more” in relation to broader sociocultural discourses on what it means to be healthy and well, versus no longer seriously ill.

Eating disorders are diagnosed by clinicians, so eating disorder recovery is also often defined clinically. This means it necessarily takes place within a sociocultural nexus laden with expert, or at least professional, expectations and ideas about which bodies are healthy [29, 31, 61, 70, 71]. Different bodies typically evoke, that is, bear the weight of [52], different attitudes and expectations. For example, those in larger bodies are typically subjected to more stringent surveillance than those in smaller bodies [72], though there is scrutiny of both “too thin” and “too fat” bodies [73]. In societies waging a protracted (and costly and losing) “war on obesity,” where “obesity management” is assumed to be absolutely vital for improving public health, we will miss the ways in which eating disorder recovery intersects with expectations for health levied at those in larger bodies—unless our analysis explicitly positions eating distress and recovery within sociocultural contexts. With emerging awareness of the barriers to treatment faced by those *without* low BMIs presenting with severe eating disorders (e.g., [74]) we are particularly called to reconsider the diagnosis-treatment-recovery link.

People in recovery may present particularized versions of recovery that align only somewhat or not at all with broader discourses, not only about “proper” health, but also, for instance, “proper” femininity, gender identity, sexuality, and/or ethnicity [20, 75]. Taking a critical feminist approach allows us to dig deeper into how such representations might play into overt and subtle exhortations for the management of health that serve to keep people small, “in control,” “nice,” and so forth. There is a difference between critiquing extant representations of recovery and critiquing the systems that make only some versions of recovery intelligible [20]. We might ask, under the current cultural conditions around health expectations, whose bodies and lives are “recoverable” [69]? For instance, if a person was never diagnosed with an eating disorder because they faced barriers to recognition on the basis of their body size [76], are they able to identify as “recovered”, particularly when they might recover into a body that is subject to particularly strong imperatives to become smaller? There is scope to explore different ways of promoting a focus on the aspects of recovery that may presently be underexplored in treatment contexts but that might align with different preferred versions of subjectivity [31].

Critical feminist work on eating recoveries invites us to consider, for example, the therapeutic and other relational networks that enable recovery to happen [53]. Given that recovery often involves going “against the grain” of cultural dictates around body size, eating, and self-control [31, 54, 70], we must look at the meanings of and practices around recoveries in context, including investigating the relational and affective ties between people in recovery and the people who support them [53]. By emphasizing that useful knowledge about eating disorders and recovery requires an understanding of people’s identities, subjectivities, and relationships to the world around them, feminist work emphasizes the value of investigating why some people might *not* recover, or at least not in “expected ways” [70, 71]. Feminists note that, since people in recovery from eating disorders use clinical and popular ideas about what recovery, eating disorders, and health are, they are likely to position themselves both in and outside of these narratives [77]. Taking a feminist approach to understanding eating disorder recovery might also involve, then, a rethinking of the very language and terminology we use to describe a state of greater well-being with respect to one’s body and practices [54, 78]. For example, the term “reclamation” may resonate better with some than the term recovery [78]. Still others might prefer to distance themselves entirely from concepts and terms around eating disorders and recoveries that do not align with their current experiences [54].

## Limitations of feminist approaches to eating disorders

Although there is great value in understanding and incorporating feminist approaches to eating disorder research and treatment, as with all approaches they are not without limitations. In line with broader critiques of feminism since its second wave, feminist work on eating disorders has not been immune to the ways in which Whiteness is a taken-for-granted norm in the broad eating disorders field [79]. Participants in eating disorder studies in *both* “mainstream” and feminist spaces tend to represent those stereotypically legitimized as having eating disorders, and particularly those diagnosed with anorexia nervosa. More research is needed that affirms the importance of conducting studies—of risk factors, symptoms, assessment, stages of the disorder, treatment, etc.—that include those with lived experiences outside of dominant young, White, thin, able-bodied, cisgender, heterosexual norms [24, 60, 80, 81]. As feminist work on eating disorders and eating distress continues to move forward, there is a need to grapple with the ways in which we have persistently caused harm in eating disorder treatment and research alike by neglecting to attend to the dis/embodied experiences of those facing racism, classism, homophobia, transphobia, ableism, and more.

Further, feminist work shares an over-focus on anorexia-like restricting, ascetic behaviours that have led to emaciation both historically and currently [52, 82, 83]. We have gained tremendous insight into thin women’s experiences through contextualising studies of restriction [see, e.g., [32, 48], but there remains a need to grapple with the lived experiences, political economies, and the cultural anthropologies of bulimia nervosa [84, 85], binge-eating disorders, and other manifestations of the spectra of eating disorders and body image problems [66]. At the same time, these are also wider omissions in eating disorder research and not specific to feminist scholarship.

## Conclusions

The major theme of this set of two articles is an invitation to researchers, scholars, clinicians, and those with lived experience of eating disorders to join us in a dialogue about the contributions, potential contributions, and shortcomings of feminist approaches. The time is right.

We have argued that, for three major reasons, feminist approaches to eating disorders need to be better understood. First, they have been ignored, misunderstood, and/or treated as a straw woman in critiques. Second, they still matter—perhaps now, more than ever—because traditional and non-traditional research methods converge in their findings that people who are marginalized, oppressed, and victimized in various sociocultural ways are at high risk for many psychological and physical illnesses, including the spectrum of eating disorders.

Third, as feminists in the field we respect and benefit from the tremendous contributions made by mainstream eating disorders and body image researchers over the past 45–50 years. Nevertheless, as feminists we believe that scientific accuracy and humility call for an acknowledgement that even our best cognitive-behavioral and neuroscientific approaches to etiology and treatment leave too much unknown, too many people suffering, and far too many excluded from the current processes that comprise identification, referral, treatment, and support. Feminist approaches can serve as useful guides for collaboration, creativity, and increased productivity in the science(s) of eating disorders. These approaches have very specific implications for attending to lived experiences through greater use of participatory research, for contextualizing treatment, prevention and advocacy by expanding the meanings of “sociocultural influences,” for situating and understanding recovery, and for diversifying methodologies.

We are not advocating for a wholesale embrace of feminist approaches to eating disorders. The field of eating disorders is too complex for that, and we reiterate feminism’s longstanding mistrust of all-embracing, totalizing theories. This principle in turn means that it does not make sense to reject or ignore feminism based on suspicions that it is irrelevant to some important aspect of the field. For example, it is the case the feminist approaches emphasize a broad array of sociocultural factors, and so does the field of eating disorders prevention [5]. It is also the case that the evidence for the prevention of anorexia nervosa is limited and equivocal [5]. Nevertheless, it does *not* follow that those committed to increasing research on understanding, preventing, and treating anorexia nervosa should ignore or demean feminist approaches. In fact, the treatment of anorexia nervosa and the phenomenon of relapse remain problematic in many ways, and feminism has a great deal to offer in thinking about empowerment of people with eating disorders and their families, the strengths and limitations of neuroscience (see, e.g., 86), and our understanding of illness and health.

Far from making eating distress silly or a passing concern or “not a real illness,” feminist work on eating disorders has, for a long time drawn our attention to how real suffering in bodies and around food is deeply linked to forms of real social suffering that have profound consequences for multiple forms of physical illness and psychological disorder. The “solution,” then, cannot be only individual, and cannot simply seek to detect, excise, and cast out the eating disorder without considering meanings made [72]. Feminist approaches insist that we situate eating distress within broader sociocultural milieu without diminishing the seriousness of suffering, such that solutions *must* be rooted in *systemic change*.

Frankly, feminist approaches call us into deeper self-reflections and conversations, all within the relational contexts of our professional, political, and personal lives, about how to better understand, prevent, and treat eating disorders. Feminist approaches invite careful, multifaceted, and indeed multivocal—and thus more socially just—considerations of the foundations of our approaches to diagnosis, treatment, and prevention, all grounded in a concern for individual and public (sociocultural) health.

We reiterate that feminism is *not* anti-scientific, and that it is not anti-scientific to interrogate, criticize, and even challenge the ways in which medicine, psychiatry, clinical psychology, neuroscience, psychopharmacology, social work, etc., are practiced. Feminism is a different paradigm within science, as well as within other disciplines, ranging from art history to medieval religious studies to economics. As a paradigm its various perspectives provide the encouragement, the tools, and the body of knowledge that invite us to question research practices themselves and the philosophies and theories that implicitly or explicitly shape how we “see” eating disorders—and thus what we don’t “see” [34]. That is, feminism provides innovative ways of knowing, doing, and being a researcher that together can open up new levels of complexity in our theories and data.

We are not asking or exhorting everyone who reads this paper to become a feminist eating disorder researcher and/or treatment provider. Rather, we invite readers to consider asking themselves about any type of resistance that might have come up while reading, and invite conversation about those spaces of tension. Our hope is to generate lively conversations about the commitments we all hold in doing this work, and to re-imagine, together, affirmative ways forward that are rooted in the lived realities of the heterogeneous group of humans, including perhaps ourselves, who experience distress in their bodies and around food. Hopefully, these conversations will arise or continue in supervised training, graduate education, the pages of journals, conferences, and other relational spaces where the field(s) of eating disorders are maintained and transformed.

## Data Availability

Not applicable.

## Footnotes

1 From LaMarre et al., 2022, this journal: “With this in mind, it is important for us to also acknowledge our own positionalities as we write this piece. Particularly important is the acknowledgment that we are all White and from Global North, English-dominant countries. Three of us are cisgender women, and one of us is a cisgender man. We are at various career stages ranging from lecturer to professor emeritus, and we come from research, prevention, and advocacy contexts and do not practice clinically. Some of us identify as having lived experience of eating disorders or distress.”.

## References

[1] LaMarre A, Levine M, Holmes S, Malson H. An open invitation to productive conversations about feminism and the spectrum of eating disorders (part 1): basic principles of feminist approaches. J Eat Disord. 2022. 10.1186/s40337-022-00532-x PMC9020002 35440011

[2] Cheng ZH, Perko VL, Fuller-Marashi L, Gau JM, Stice E (2019). Ethnic differences in eating disorder prevalence, risk factors, and predictive effects of risk factors among young women. Eat Behav.

[3] Nagata JM, Ganson KT, Austin SB (2020). Emerging trends in eating disorders among sexual and gender minorities. Curr Opin Psychiatry.

[4] Jasper K, Smolak L, Levine MP (2015). Feminist therapy. The Wiley handbook of eating disorders, volume II: assessment, prevention, treatment, policy, and future directions.

[5] Levine MP, Smolak L (2021). The prevention of eating problems and eating disorders: theories, research, and applications.

[6] Piran N, Piran N, Levine MP, Steiner-Adair C (1999). The reduction of preoccupation with body weight and shape in schools: A feminist approach. Preventing eating disorders: a handbook of interventions and special challenges.

[7] Piran N, Smolak L, Levine MP (2015). A feminist perspective on the prevention of eating disorders. The Wiley handbook of eating disorders, volume II: assessment, prevention, treatment, policy, and future directions.

[8] Lock J (2009). What is the best way to treat adolescents with anorexia nervosa?. Eat Disord.

[9] Houston Grey S. A perfect loathing: the feminist expulsion of the eating disorder. KB J. 2011;7(2). http://kbjournal.org/grey

[10] Wooley S, Fallon P, Katzman MA, Wooley SC (1994). Sexual abuse and eating disorders: the concealed debate. Feminist perspectives on eating disorders.

[11] Boskind-Lodahl M (1976). Cinderella's stepsisters: a feminist perspective on anorexia nervosa and bulimia. Signs.

[12] Chernin K (1985). The hungry self: women, eating and identity.

[13] Sesan R, Fallon P, Katzman MA, Wooley SC (1994). Feminist inpatient treatment for eating disorders: an oxymoron. Feminist perspectives on eating disorders.

[14] Orbach S (1978). Fat is a feminist issue: the anti-diet guide to permanent weight loss.

[15] Orbach S, Emmett SW (1985). Visibility/invisibility: social considerations in anorexia nervosa—a feminist perspective. Theory and treatment of anorexia nervosa and bulimia.

[16] Fallon P, Katzman MA, Wooley SC, editors. Feminist perspectives on eating disorders. Guilford; 1994.

[17] Lester R (2019). Famished: eating disorders and failed care in America.

[18] Skoger UE, Magnussen E (2015). What makes feminist knowledge legitimate for therapists? A study of Swedish child psychotherapists. Feminism Psychol..

[19] Bloom C, Gitter A, Gutwill S, Kogel L, Zaphiropoulos L, Women's Therapy Center Therapy Institute. Eating problems: a feminist psychoanalytic treatment model. Basic Books, 1994.

[20] Holmes S (2017). “My anorexia story”: girls constructing narratives of identity on YouTube. Cult Stud.

[21] Holmes S (2016). “Blindness to the obvious”? Treatment experiences and feminist approaches to eating disorders. Feminism Psychol.

[22] Holmes S, Drake S, Odgers K, Wilson J (2017). Feminist approaches to anorexia nervosa: a qualitative study of a treatment group. J Eat Disord.

[23] Thapliyal P, Hay P, Conti J (2018). Role of gender in the treatment experiences of people with an eating disorder: a metasynthesis. J Eat Disord.

[24] Katzman MA, Lee S (1997). Beyond body image: the integration of feminist and transcultural theories in the understanding of self starvation. Int J Eat Disord.

[25] Duffy ME, Henkel KE, Earnshaw VA (2016). Transgender clients’ experience of eating disorder treatment. J LGBTQ Issues Counsel.

[26] Hartman-Munick SM, Silverstein S, Guss CE, Lopez E, Calzo JP, Gordon AR (2021). Eating disorder screening and treatment experiences in transgender and gender diverse young adults. Eat Behav.

[27] Gremillion H (2003). Feeding anorexia: gender and power at a treatment center.

[28] Boughtwood D, Halse C (2010). Other than obedient: girls’ constructions of doctors and treatment regimes for anorexia nervosa. J Commun Appl Psychol.

[29] Saukko P (2008). The anorexic self: a personal, political analysis of a diagnostic discourse.

[30] Brown-Bowers A, Ward A, Cormier N (2017). Treating the binge or the (fat) body? Representations of fatness in a gold standard psychological treatment manual for binge eating disorder. Health.

[31] Malson H, Bailey L, Clarke S, Treasure J, Anderson G, Kohn M (2011). Un/imaginable future selves: a discourse analysis of in-patients' talk about recovery from an “eating disorder”. Eur Eat Disord Rev.

[32] Warin M (2009). Abject relations: everyday worlds of anorexia.

[33] Geller J, Maiolino N, Samson L, Srikameswaran S (2021). Is experiencing care as collaborative associated with enhanced outcomes in inpatient eating disorders treatment?. Eat Disord.

[34] Hussain A, Dar M, Ganson KT. A post-structural feminist analysis of eating disorders intervention research. Affilia. 2021; advance online publication. 10.1177/08861099211062942

[35] National Institute for Health and Care Excellence (NICE). Eating disorders: recognition and treatment. 2017/updated 2020. Retrieved from https://www.nice.org.uk/guidance/NG69 33689256

[36] Gelso CJ, Kivlighan DM, Markin RD (2018). The real relationship and its role in psychotherapy outcome: a meta-analysis. Psychotherapy.

[37] Smolak L, Levine MP, Smolak L, Levine MP (2015). Toward an integrated biopsychosocial model of eating disorders. The Wiley handbook of eating disorders, volume II: assessment, prevention, treatment, policy, and future directions.

[38] Henderson L-T, Esposito J (2019). Using others in the nicest way possible: on colonial and academic practice(s), and an ethic of humility. Qualit Inq..

[39] Hooks B (1984). Feminist theory: from margin to center.

[40] Pugh M (2020). Understanding ‘Ed’: a theoretical and empirical review of the internal eating disorder ‘voice’. Psychother Sect Rev..

[41] Hampshire K, Tierney S, Varese F, Haddock G, Saeidi S, Fox JRE (2020). The development and assessment of a scale to measure the experience of an anorexic voice in anorexia nervosa. Clin Psychol Psychother.

[42] Higbed L, Fox JRE (2010). Illness perceptions in anorexia nervosa: a qualitative investigation. Br J Clin Psychol.

[43] Tierney S, Fox JRE (2010). Living with the anorexic voice: a thematic analysis. Psychol Psychother.

[44] Pugh M (2016). The internal ‘anorexic voice’: a feature or fallacy of eating disorders. Adv Eat Disord.

[45] White M. Narrative practice: continuing the conversations (Denborough D, editor). W.W. Norton; 2011.

[46] Saukko P, Malson H, Burns M (2009). A critical discussion of normativity in discourses on eating disorders. Critical feminist approaches to eating dis/orders.

[47] Gill R (2008). Culture and subjectivity in neoliberal and postfeminist times. Subjectivity.

[48] Malson H (1998). The thin woman: feminism, post-structuralism, and the social psychology of anorexia nervosa.

[49] Katzman MA (1997). Getting the difference right: it’s power not gender that matters. Eur Eat Disord Rev.

[50] Nasser M, Malson H, Malson H, Burns M (2009). Beyond Western dis/orders: thinness and self-starvation of other-ed women. Critical feminist approaches to eating dis/orders.

[51] Bordo S, Diamond I, Quinby L (1988). Anorexia nervosa: psychopathology as the crystallization of culture. Feminism and Foucault: reflections on resistance.

[52] Bordo S (1993). Unbearable weight: feminism, Western culture, and the body.

[53] LaMarre A, Rice C (2021). The eating disorder recovery assemblage: collectively generating possibilities for eating disorder recovery. Feminism Psychol.

[54] LaMarre A, Rice C (2021). Recovering uncertainty: eating disorder recovery in context. Cult Med Psychiatry.

[55] Ali K, Farrer L, Fassnacht DB, Gulliver A, Bauer S, Griffiths KM (2017). Perceived barriers and facilitators towards help-seeking for eating disorders: a systematic review. Int J Eat Disord.

[56] Becker AE, Franko DL, Speck A, Herzog DB (2003). Ethnicity and differential access to care for eating disorder symptoms. Int J Eat Disord.

[57] Cachelin FM, Rebeck R, Veisel C, Striegel-Moore RH (2001). Barriers to treatment for eating disorders among ethnically diverse women. Int J Eat Disord.

[58] Boughtwood D, Halse C (2008). Ambivalent appetites: dissonances in social and medical constructions of anorexia nervosa. J Commun Appl Soci Psychol.

[59] Malson H, Finn DM, Treasure J, Clarke S, Anderson G (2004). Constructing ‘the eating disordered patient’: a discourse analysis of accounts of treatment experiences. J Commun Appl Soc Psychol.

[60] Piran N. Journeys of embodiment at the intersection of body and culture: the developmental theory of embodiment. Academic Press; 2017.

[61] LaMarre A, Rice C (2016). Embodying critical and corporeal methodology: digital storytelling with young women in eating disorder recovery. Forum: Qualit Soc Res..

[62] Saunders J, Eaton A (2018). Social comparisons in eating disorder recovery: using PhotoVoice to capture the sociocultural influences on women's recovery. Int J Eat Disord.

[63] Levy G, Halse C, Wright J (2016). Down the methodological rabbit hole: thinking diffractively with resistant data. Qualit Res.

[64] Barad K (2007). Meeting the university halfway: quantum physics and the entanglement of matter and meaning.

[65] Deleuze G. The logic of sense. In Lester M, Stivale C, translators; Boundas CV, editor. Columbia University Press; 1990.

[66] Wolf N (1991). The beauty myth: how images of beauty are used against women.

[67] Bardone-Cone A, Harney MB, Maldonado CR, Lawson MA, Robinson P, Smith R, Tosh A (2010). Defining recovery from an eating disorder: conceptualization, validation, and examination of psychosocial functioning and psychiatric comorbidity. Beh Res Ther..

[68] Bardone-Cone AM, Hunt RA, Watson HJ (2018). An overview of conceptualizations of eating disorder recovery, recent findings, and future directions. Curr Psychiatry Rep.

[69] LaMarre A, Rice C, Bear M. (2015). Unrecoverable? Prescriptions and possibilities for eating disorder recovery. In: Khanlou N, Pilkington F, editors. Women's mental health: advances in mental health and addiction. Springer; 2015. p. 145–160.

[70] Musolino C, Warin M, Wade T, Gilchrist P (2016). Developing shared understandings of recovery and care: a qualitative study of women with eating disorders who resist therapeutic care. J Eat Disord.

[71] Musolino CM, Warin M, Gilchrist P (2020). Embodiment as a paradigm for understanding and treating SE-AN: locating the self in culture. Front Psychiatry.

[72] Trainer S, Brewis A, Wutich A (2007). Eating in the Panopticon: Surveillance of food and weight before and after bariatric surgery. Med Anth.

[73] Lupton D. Fat. Routledge; 2013.

[74] Harrop EN, Mensinger JL, Moore M, Lindhorst T (2021). Restrictive eating disorders in higher weight persons: A systematic review of atypical anorexia nervosa prevalence and consecutive admission literature. Int J Eat Disord.

[75] LaMarre A, Rice C (2017). Hashtag recovery: #Eating Disorder Recovery on Instagram. Soc Sci.

[76] Lebow J, Sim LA, Kransdorf LN (2015). Prevalence of a history of overweight and obesity in adolescents with restrictive eating disorders. J Adol Health.

[77] Shohet M (2018). Beyond the clinic? Eluding a medical diagnosis of anorexia through narrative. Transcult Psychiatry.

[78] Conti JE (2018). Recovering identity from anorexia nervosa: women's constructions of their experiences of recovery from anorexia nervosa over 10 years. J Constructivist Psychology.

[79] Burke NL, Schaefer LM, Hazzard VM, Rodgers RF (2020). Where identities converge: the importance of intersectionality in eating disorders research. Int J Eat Disord.

[80] Thompson BW (1992). “A way outa no way”: eating problems among African-American, Latina, and White women. Gender Society.

[81] Thompson BW (1994). A hunger so wide and so deep.

[82] Bruch H (1978). The golden cage: the enigma of anorexia nervosa.

[83] Brumberg J. Fasting girls: the emergence of anorexia as a modern disease. Harvard University Press; 1988. 10.1126/science.240.4855.1061 17731731

[84] Burns M (2004). Eating like an ox: feminism and dualistic constructions of bulimia and anorexia. Feminism Psychol.

[85] Burns M, Malson H, Burns M (2009). Bodies as (im)material? Bulimia and body image discourse. Critical feminist approaches to eating dis/orders.

[86] Schmitz S, Höppner G (2014). Neurofeminism and feminist neurosciences: a critical review of contemporary brain research. Front in Human Neurosci.

